# Prevalence, features, and explanations of missed and misinterpreted pancreatic cancer on imaging: a matched case–control study

**DOI:** 10.1007/s00261-022-03671-6

**Published:** 2022-09-21

**Authors:** Sanne A. Hoogenboom, Megan M. L. Engels, Anthony V. Chuprin, Jeanin E. van Hooft, Jordan D. LeGout, Michael B. Wallace, Candice W. Bolan

**Affiliations:** 1 Department of Gastroenterology and Hepatology, Mayo Clinic, 4500 San Pablo Road South, Jacksonville, FL 32224 USA; 2 Department of Radiology, Mayo Clinic, 4500 San Pablo Road South, Jacksonville, FL 32224 USA; 3 Department of Gastroenterology and Hepatology, Amsterdam Gastroenterology Endocrinology Metabolism, Amsterdam UMC, University of Amsterdam, Meibergdreef 9, 1105 AZ Amsterdam, Netherlands; 4 Department of Gastroenterology and Hepatology, Leiden University Medical Center, Albinusdreef 2, 2333 ZA Leiden, Netherlands; 5 Department of Gastroenterology and Hepatology, Sheikh Shakhbout Medical City, PO Box 11001, Abu Dhabi, UAE; 6 Khalifa University School of Medicine, PO Box 127788, Abu Dhabi, UAE

**Keywords:** Pancreatic ductal adenocarcinoma, Computed tomography, Magnetic resonance imaging, Case–control studies

## Abstract

**Purpose:**

To characterize the prevalence of missed pancreatic masses and pancreatic ductal adenocarcinoma (PDAC)-related findings on CT and MRI between pre-diagnostic patients and healthy individuals.

**Materials and methods:**

Patients diagnosed with PDAC (2010–2016) were retrospectively reviewed for abdominal CT- or MRI-examinations 1 month—3 years prior to their diagnosis, and subsequently matched to controls in a 1:4 ratio. Two blinded radiologists scored each imaging exam on the presence of a pancreatic mass and secondary features of PDAC. Additionally, original radiology reports were graded based on the revised RADPEER criteria.

**Results:**

The cohort of 595 PDAC patients contained 60 patients with a pre-diagnostic CT and 27 with an MRI. A pancreatic mass was suspected in hindsight on CT in 51.7% and 50% of cases and in 1.3% and 0.9% of controls by reviewer 1 (*p* < .001) and reviewer 2 (*p* < .001), respectively. On MRI, a mass was suspected in 70.4% and 55.6% of cases and 2.9% and 0% of the controls by reviewer 1 (*p* < .001) and reviewer 2 (*p* < .001), respectively. Pancreatic duct dilation, duct interruption, focal atrophy, and features of acute pancreatitis is strongly associated with PDAC (*p* < .001). In cases, a RADPEER-score of 2 or 3 was assigned to 56.3% of the CT-reports and 71.4% of MRI-reports.

**Conclusion:**

Radiological features as pancreatic duct dilation and interruption, and focal atrophy are common first signs of PDAC and are often missed or unrecognized. Further investigation with dedicated pancreas imaging is warranted in patients with PDAC-related radiological findings.

**Graphical abstract:**

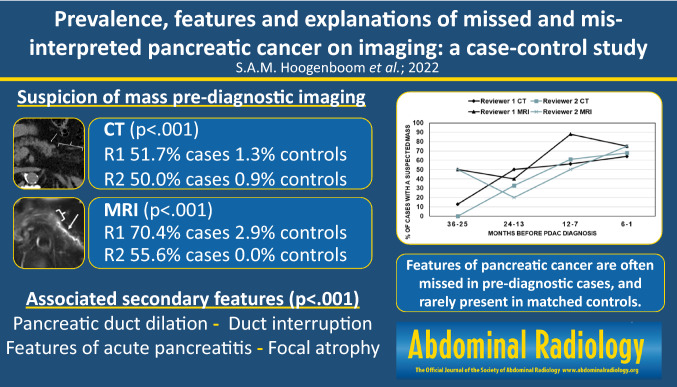

**Supplementary Information:**

The online version contains supplementary material available at 10.1007/s00261-022-03671-6.

## Introduction

The prognosis of pancreatic ductal adenocarcinoma (PDAC) is among the poorest of all cancers with a 5-year survival rate of only 9% [1]. Improvement in survival is constrained, given most patients are diagnosed with PDAC in an advanced stage due to the late onset of symptoms. Since the 5-year survival rate of patients with localized tumors (< 1 cm) is considerably better (71%—84%), early detection is imperative to improve the prognosis and survival of PDAC [2, 3].

PDAC arises from ductal epithelial cells and precursor lesions are common in the general elderly population, including pancreatic intraepithelial neoplasia (PanIN), intraductal pancreatic mucinous neoplasia (IPMN), and mucinous cystic neoplasia [4, 5]. Although these precursor lesions predominantly harbor low-grade dysplasia, a fraction will evolve into high-grade dysplasia and these are considered immediate precursors of PDAC [6]. The estimated time of progression from the initiation of PanIN to PDAC and from PDAC to potential metastasis is 12 and 7 years, respectively [7, 8]. Considering this slow progression, there is a notable window of opportunity to detect early-stage PDAC or even precursor lesions with high-grade dysplasia.

The increasing utilization of cross-sectional-imaging, including multidetector computed tomography (CT) and magnetic resonance imaging (MRI), poses a unique opportunity for incidental detection of early-stage PDAC in patients undergoing abdominal imaging for nonspecific or unrelated symptoms. Although both MRI and CT are sensitive methods for detecting patients with *symptomatic* PDAC, the sensitivity of these modalities is limited in asymptomatic patients [9, 10]. Subtle pancreatic masses or related features may not always be perceived, for instance when imaging is performed in an emergency setting or for indications unrelated to the pancreas, or in hospitals without specific pancreatic expertise.

Interestingly, various studies have reported the presence of secondary imaging features related to PDAC up to 5 years before the eventual diagnosis, such as pancreatic duct dilation or interruption [11–19]. It remains unclear if these features are specific for pre-diagnostic PDAC or can be observed in individuals who are not diagnosed with PDAC in subsequent years, as only studies with limited sample sizes compared early imaging features between cases and controls [17, 18]. Therefore, the aim of this study was to assess and compare the prevalence of suspected pancreatic masses and secondary signs of PDAC on CT and MRI among two groups: patients diagnosed with PDAC within 3 years of an imaging exam, and matched individuals who were not diagnosed with PDAC in subsequent years. In addition, possible explanations for missing or misinterpreting secondary signs and masses were evaluated in the case group. The results of this study are valuable for raising awareness of specific secondary signs of PDAC among clinicians and radiologists, for a better understanding of the radiological errors underlying missing or misinterpreted secondary signs, and for emphasizing the importance of thorough review for these features on all imaging exams.

## Materials and methods

### Study design

A single-center, retrospective, case–control study was conducted among PDAC patients with a pre-diagnostic CT- and/or MRI-exam 3 years prior to diagnosis, and healthy individuals. Part of the subjects, i.e., 32 cases and 117 control subjects, were used in a previously published study assessing the presence of pancreatic steatosis on non-enhanced CT in patients with pre-diagnostic PDAC [20]. This current study focuses on distinct imaging features of pre-diagnostic PDAC on CT and MRI, instead of pancreatic steatosis on CT only. The study protocol was approved, with waiver of consent, by the Institutional Review Board of Mayo Clinic Florida (18–002403).

### Case selection

Electronic medical records of all patients with histopathologically confirmed PDAC, diagnosed between 2010 and 2016, were retrospectively reviewed. Patients who underwent CT- and/or MRI-examinations 1 month–3 years prior to the diagnosis of PDAC were included in the study as cases. The date of PDAC diagnosis was defined as the date of cytological or histological confirmation of the disease. If a subject had multiple CT- or MRI-exams in that time frame, the study nearest in time to diagnosis was selected. All imaging protocols of pre-diagnostic CTs and MRIs were included, to reduce selection bias and mimic real-world practice. Patients were excluded when the pre-diagnostic imaging report clearly noted a pancreatic mass; imaging was conducted within 4 weeks after abdominal surgery; PDAC was a recurrence; or patients had a previous history of pancreatic surgery. Clinical variables of age, gender, tumor size at diagnosis, and time between imaging and diagnosis were retrospectively collected for cases and controls.

### Control selection

Controls were selected in a 4:1 ratio to cases to increase the statistical power of the study. Controls were defined as patients with CT- or MRI-examinations who did not develop PDAC within 3 years after imaging. Control subjects were matched to cases by gender, age, imaging modality (CT or MR), usage of contrast, date of imaging (± 3 months), and randomly selected from the internal radiologic database Illuminate Insight™ [21]. Medical records were reviewed to ascertain that controls had at least 3 years of clinical follow-up after the selected imaging in which they did not develop PDAC. Controls were excluded if lost to follow-up within 3 years after imaging; imaging was conducted within 4 weeks after abdominal surgery; or a history of pancreatic malignancy or pancreatic surgery was reported.

### Imaging analysis

Two board certified abdominal radiologists (C.W.B. and J.D.L), with 10 and 5 years of experience, respectively, reviewed all CT- and MR-imaging exams blinded to case and control status. Imaging exams were independently reviewed on a dedicated workstation using Visage 7.1 Picture Archiving and Communication System (PACS). The exams were reassessed on radiological features of PDAC, including the presence of a mass, (focal) atrophy and pancreatitis, perivascular soft tissue, and pancreatic duct (PD) dilation (> 3 mm) or interruption (Supplemental material 1). When a mass was suspected, the confidence of the suspicion (high or low), size of the mass, and TNM stage according to the American Joint Committee on Cancer staging (8th edition) (22) were reported.

### Second reassessment

After the blinded reassessment, reviewer 1 was unblinded for case and control status and the reassessment results from reviewer 2. In addition, the original radiology report, the indication of imaging and the location of the eventual tumor were provided. Reviewer 1 scored all available pre-diagnostic imaging reports according to the 2016 revised RADPEER criteria, a peer review tool developed to assess diagnostic performance and identify areas for improvement on a 3-point scale [22] (Supplemental material 1). A score of 1 indicates agreement with the previous reader, a score of 2 denotes a discrepancy in reading, yet categorized as an understandable miss, and a score of 3 indicates a discrepancy in interpretation that should have been made. All images with a RADPEER score of 2 or 3 were then assessed for possible explanations regarding missed or misinterpreted radiological features of PDAC, according to the classification of errors by Kim and Mansfield [23] (Supplemental material 4). These include technical limitations, perceptual errors (i.e., abnormality is simply not seen) or cognitive errors (i.e., abnormality is visually detected but incorrectly interpreted), and insufficient communication and/or follow-up.

### Statistical analysis

Microsoft Excel was used for data management and JMP Pro (v14.1.0 SAS Institute Inc, North Carolina, USA) for statistical analysis. Continuous variables were reported as mean (standard deviation; SD) or median (range or interquartile range; IQR) and compared by either Student *T* test or Mann–Whitney *U* test. Categorical variables were presented as frequencies with percentages and compared using the Chi-square test or Fisher’s exact test. Two-sided *p* values less than 0.05 were considered statistically significant. The interobserver agreement between the reviewers was calculated using Cohen’s kappa (*k*); values ≤ 0 indicate no agreement, 0.01–0.20 indicate none to slight, 0.21–0.40 fair, 0.41–0.60 moderate, 0.61–0.80 substantial, and 0.81–1.00 almost perfect agreement. The sensitivity and specificity of secondary signs of PDAC were calculated for both reviewers and reported with a 95% confidence interval.

## Results

**Fig. 1:**
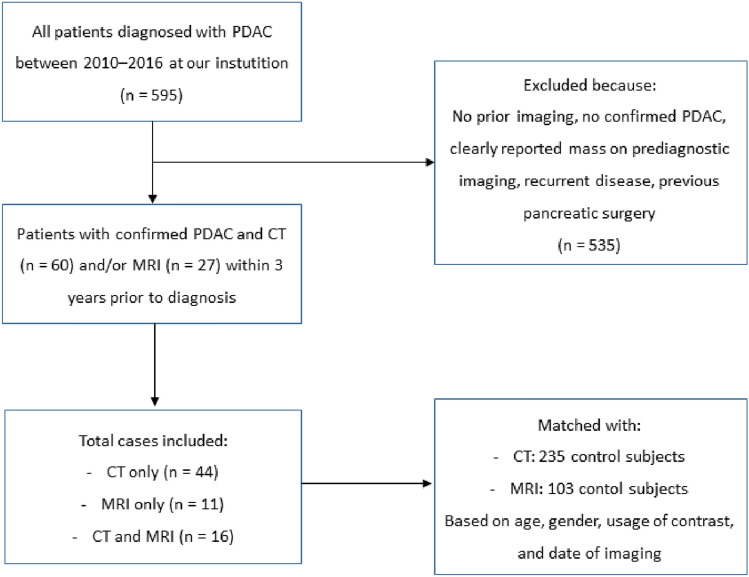
Flow chart of patient inclusion

Between 2010 and 2016, 595 patients were diagnosed with PDAC at our institution (Fig. 1). Seventy-one (11.9%) patients met the inclusion criteria and underwent cross-sectional imaging within 3 years of diagnosis. Of 71 patients, 16 underwent both CT and MRI. A total of 60 CT- and 27 MRI-examinations were evaluated. The indications for imaging of cases and controls can be found in Supplemental material 2 (CT) and Supplemental material 3 (MRI).

### CT

All 60 cases, who underwent CT-imaging in the 3 years prior to PDAC diagnosis, were matched with 235 controls. Baseline characteristics are presented in Table 1. During reassessment, at least one reviewer suspected a pancreatic mass in 37/60 cases, and 26/37 of these suspicions were with high confidence. Median mass size was 21 mm (range 8–50 mm), 11 were staged on imaging as T1, 13 as T2, 1 as T3, and 1 as T4.

**Table 1:** Baseline characteristics

	Cases (*n* = 60)	Controls (*n* = 235)	*p* value
CT			
Female, *n* (%)	23 (38.3)	92 (39.1)	1.00
Age at diagnosis, mean (SD)	68.5 (10.9)	–	
Age at imaging, mean (SD)	67.5 (10.8)	67.2 (10.5)	.86
Time between imaging and diagnosis, months, median (IQR)	7.9 (4.7–14.7)	–	
Tumor size (mm) at diagnosis, median (IQR)	28 (21–33)	–	
CT protocol			
• CT without contrast, *n* (%)	21 (35.0)	79 (33.6)	.98
•CT with IV contrast, *n* (%)	26 (43.3)	105 (44.7)	
•CT with and without IV contrast, *n* (%)	13 (21.7)	51 (21.7)	
CT slice thickness, median (IQR)	5 (5–5)	5 (5–5)	.37
Imaging institution			
•Internal, *n* (%)	20 (33.3)	235 (100)	< .001
•External, *n* (%)	40 (66.7)	–	

	Cases (*n* = 27)	Controls (*n* = 103)	*p* value
MRT			
Female, *n* (%)	15 (55.6)	59 (57.3)	1.00
Age at diagnosis, mean (SD)	68.7 (7.8)	–	
Age at imaging, mean (SD)	67.9 (7.7)	67.3 (7.2)	.72
Time between imaging and diagnosis, months, median (IQR)	7.4 (4.3–13.4)	–	
Tumor size (mm) at diagnosis, median (IQR)*	25 (21–28)	–	
MRI protocol			
MRI with and without contrast, *n* (%)	14 (51.9)	79 (76.7)	.01
MRI + MRCP with and without contrast, *n* (%)	9 (33.3)	22 (21.4)	
MRI without contrast, *n* (%)	2 (7.4)	2 (1.9)	
MRCP only without contrast, *n* (%)	2 (7.4)	–	
DWI available, *n* (%)	19 (73.1)	100 (97.1)	< .001
Magnet strength (Tesla)^a^			
•0.7, *n* (%)	1 (3.8)	–	.17
•1.5, *n* (%)	18 (69.2)	81 (78.6)	
•3, *n* (%)	7 (26.9)	22 (21.4)	
Imaging institution			
•Internal, *n* (%)	16 (59.3)	103 (100.0)	< .001
•External, *n* (%)	11 (40.7)	–	

*CT* computed tomography, *MRI* magnetic resonance imaging, *MRCP* magnetic resonance cholangio-pancreatography, *SD* standard deviation, *IQR* interquartile range, *SD* standard deviation, *DWI* diffusion-weighted imaging

*Tumor size on diagnostic imaging was only available in 13/27 cases

^a^Magnet strength missing for 1/27 cases

**Fig. 2:**
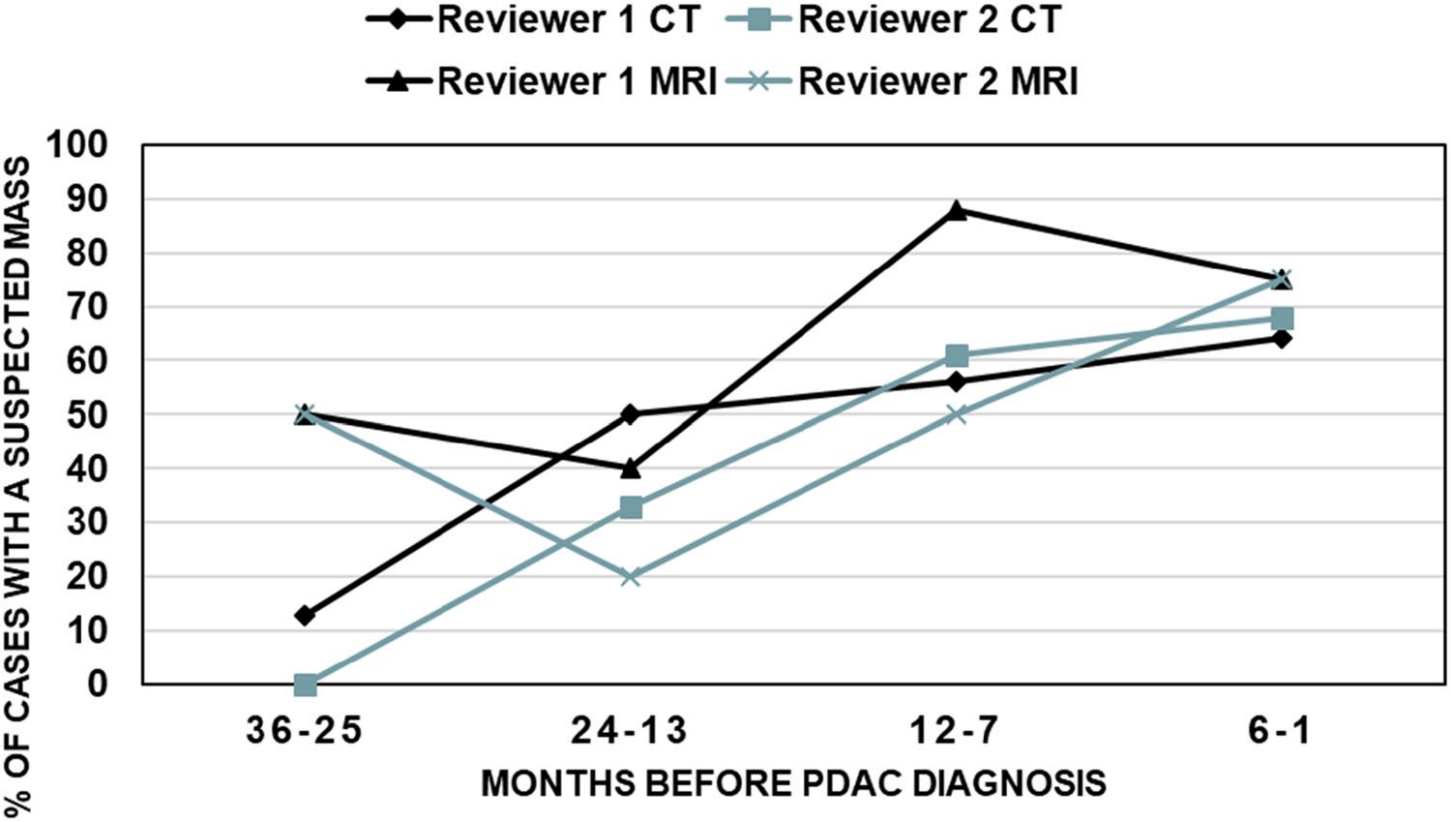
Suspected masses according to the time before PDAC diagnosis. This figure indicates the percentage of masses found by the reviewers on pre-diagnostic imaging, grouped by months prior to PDAC diagnosis. The graph shows an upwards trend toward more masses being recognized closer to diagnosis

A pancreatic mass was suspected in 31/60 cases and in 3/235 controls by reviewer 1 (*p* < 0.001). Reviewer 2 suspected a mass in 30/60 cases and 2/235 controls (*p* < 0.001). The interobserver agreement between the two radiologists was *substantial* (*k* = 0.69). The closer the pre-diagnostic imaging occurred to the eventual PDAC diagnosis, the higher the percentage of suspected masses by both reviewers (Fig. 2). A detailed summary of the reassessment can be found in Table 2.

**Table 2:** Blinded reassessment CT- and MR- imaging

CT						
	Reviewer 1			Reviewer 2		
	Cases (*n* = 60)	Controls (*n* = 235)	*p* value	Cases (*n* = 60)	Controls (*n* = 235)	*p* value
Normal pancreas, *n* (%)	9 (15.0)	158 (67.2)	< .001	11 (18.3)	173 (73.6)	< .001
Signs of acute pancreatitis, *n* (%)	6 (10.0)	3 (1.3)	0.003	7 (11.7)	3 (1.3)	< .001
• Diffuse	4 (66.7)	2 (66.7)	> .99	4 (57.1)	1 (33.3)	> .99
• Focal	2 (33.3)	1 (33.3)		3 (42.9)	2 (66.7)	
Signs of chronic pancreatitis, *n* (%)	–	1 (0.4)	> .99	–	1 (0.3)	> .99
Pancreatic cystic lesion, *n* (%)	10 (16.7)	14 (6.0)	0.014	8 (13.3)	13 (5.5)	0.048
Pancreatic duct dilation (> 3 mm), *n* (%)	12 (20.0)	–	< .001	15 (25.0)	1 (0.4)	< .001
Pancreatic duct Interruption, *n* (%)	12 (20.0)	–	< .001	25 (41.7)	–	< .001
Parenchymal atrophy, *n* (%)	34 (56.7)	65 (27.7)	< .001	24 (40.0)	46 (19.6)	< .001
• Diffuse	18 (52.9)	65 (100)	< .001	13 (54.2)	45 (97.8)	< .001
• Focal	16 (47.1)	–		11 (45.8)	1 (2.2)	
Pancreatic mass, *n* (%)	31 (51.7)	3 (1.3)	< .001	30 (50.0)	2 (0.9)	< .001
• High confidence	19 (61.3)	–	0.076	20 (66.7)	–	.13
• Low confidence	12 (38.7)	3 (1.3)		10 (33.3)	2 (100)	
Perivascular soft tissue	7 (11.7)	1 (0.4)	< .001	8 (15.4)	–	< .001
CBD diameter, median (range)	4 (26)	3 (14)	0.310	3 (27)	3 (16)	.03

MRI						
	Reviewer 1			Reviewer 2		
	Cases (*n* = 27)	Controls (*n* = 103)	*p* value	Cases (*n* = 27)	Controls (*n* = 103)	*p* value
Normal pancreas, *n* (%)	2 (7.4)	61 (59.2)	< .001	1 (3.7)	61 (59.2)	< .001
Signs of acute pancreatitis, *n* (%)	4 (14.8)	–	0.002	2 (7.4)	–	.04
•Diffuse	–	–	N/A	–	–	N/A
•Focal	4 (100.0)	–		2 (100.0)	–	
Signs of chronic pancreatitis, *n* (%)	3 (11.1)	1 (1.0)	0.028	1 (3.7)	1 (1.0)	.37
Pancreatic cystic lesion, *n* (%)	11 (40.7)	41 (39.8)	0.93	14 (51.9)	40 (38.9)	.22
Pancreatic duct dilation (> 3 mm), *n* (%)	11 (40.7)	–	< .001	13 (48.1)	–	< .001
Pancreatic duct interruption, *n* (%)	12 (44.4)	–	< .001	14 (51.9)	–	< .001
Parenchymal atrophy, *n* (%)	19 (70.4)	24 (23.3)	< .001	17 (63.0)	16 (15.5)	< .001
•Diffuse	10 (52.6)	24 (100.0)	< .001	7 (41.1)	16 (100.0)	< .001
•Focal	9 (47.4)	–		10 (48.9)	–	
Pancreatic mass, *n* (%)	19 (70.4)	3 (2.9)	< .001	15 (55.6)	–	< .001
•High confidence	14 (73.7)	1 (33.3)	0.227	12 (80.0)	–	N/A
•Low confidence	5 (26.3)	2 (66.7)		3 (20.0)	–	
Perivascular soft tissue	3 (11.1)	–	0.008	*	*	
CBD diameter in mm, median (range)	4 (11)	5 (10)	0.021	5.5 (9)	4.0 (9)	.01

*CT* computed tomography, *MRI* magnetic resonance imaging, *CBD* common bile duct

*Data missing from second reviewer

### MRI

In total, 27 cases with an MRI prior to diagnosis were identified and matched with 103 controls. Baseline characteristics are presented in Table 1. The reviewers suspected a pancreatic mass in 21/27 cases, of which 17 were with high confidence by at least one of the reviewers. Median mass size was 18 mm (range 8–43 mm). The staging of the 17 masses with high suspicion were as follows: T1 (*n* = 11), T2 (*n* = 3), T3 (*n* = 1), and T4 (*n* = 1) and metastatic disease (*n* = 1).

Reviewer 1 suspected a mass in 19/27 of the cases and 3/103 of the controls (*p* < 0.001), and reviewer 2 in 15/27 of the cases and none of the controls *(p* < 0.001). The interobserver agreement between the reviewers was *substantial* (*k* = 0.66). Consistent with the reassessment of the CT-exams, a mass was more often suspected when the imaging was conducted closer in time to diagnosis (Fig. 2). A detailed summary of the reassessment findings can be found in Table 2.

#### Diagnostic value of secondary features on MRI and CT

The sensitivity and specificity of possible secondary features of PDAC on MRI and CT, including pancreatic duct dilation, pancreatic duct interruption, parenchymal and focal atrophy, acute pancreatitis and perivascular soft tissue, are reported in Table 3.

**Table 3:** Sensitivity and specificity of pancreatic features found on CT and MRI

	Reviewer 1		Reviewer 2		*k*
	Sensitivity % (95% CI)	Specificity % (95% CI)	Sensitivity % (95% CI)	Specificity % (95% CI)	
Pancreatic duct dilation(> 3 mm)					
MRI	11/27 40.7 (22.4–61.2)	103/103 100.0 (96.5–100.0)	13/27 48.2 (28.7–68.1)	103/103 100.0 (96.5–100.0)	0.76
CT	12/60 20.0 (10.8–32.3)	235/235 100.0 (98.4–100.0)	15/60 25.0 (14.7–37.9)	234/235 99.6 (97.7–100.0)	0.78
Pancreatic duct interruption					
MRI	12/27 44.4 (25.5–64.7)	103/103 100.0 (96.5–100.0)	14/27 51.9 (32.0–71.3)	103/103 100.0 (96.5–100.0)	0.91
CT	12/60 20.0 (10.8–32.3)	235/235 100.0 (98.4–100.0)	25/60 41.7 (29.1–55.1)	235/235 100.0 (98.4–100.0)	0.51
Parenchymal atrophy					
MRI	19/27 70.4 (49.8–86.3)	79/103 76.7 (67.3–84.5)	17/27 63.0 (42.4–80.1)	87/103 84.5 (76.0–90.1)	0.63
CT	34/60 56.7 (43.2–69.4)	170/235 72.3 (66.2–78.0)	24/60 40.0 (27.6–52.5)	189/235 80.4 (74.8–85.3)	0.40
Focal atrophy					
MRI	9/27 33.3 (16.5–54.0)	103/103 100.0 (96.5–100.0)	10/27 37.0 (19.4–57.6)	103/103 100.0 (96.5–100.0)	0.94
CT	16/60 26.7 (16.1–39.7)	235/235 100.0 (98.4–100.0)	11/60 18.3 (9.5–30.4)	234/235 99.6 (97.7–100.0)	0.58
Acute pancreatitis					
MRI	4/27 14.8 (4.2–33.7)	103/103 100.0 (96.5–100.0)	2/27 7.4 (0.9–24.3)	103/103 100.0 (96.5–100.0)	0.66
CT	6/60 10.0 (3.8–20.5)	232/235 98.7 (96.3–99.7)	7/60 11.7 (4.8–22.6)	232/235 98.7 (96.3–99.7)	0.73
Perivascular soft tissue					
MRI	3/27 11.1 (2.35–29.2)	103/103 100.0 (96.5–100.0)	*	*	*
CT	7/60 11.7 (4.8–22.6)	234/235 99.6 (97.7–99.9)	8/60 13.3 (5.9–24.6)	235/235 100.0 (98.4–100.0)	0.61

*CI* confidence interval, *MRI* magnetic resonance imaging, *CT* computed tomography, *k* Cohen’s Kappa for interobserving agreement

*Data missing

#### RADPEER score and radiological errors

**Fig. 3:**
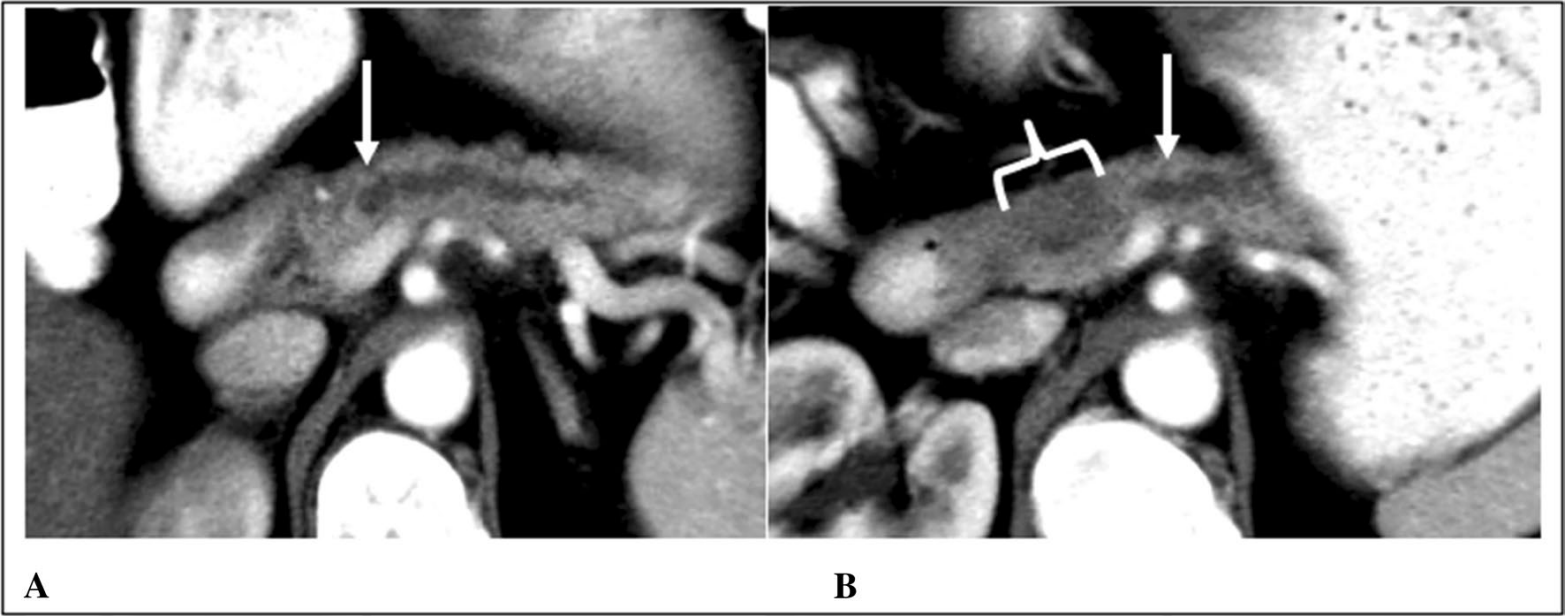
RADPEER 3 Score on CT—underread and missed findings. A case of a 48-year old female with epigastric pain. Axial postcontrast venous phase CT images (**A**, **B**) demonstrate pancreatic duct dilation (arrow) with abrupt termination in the head at a 20 mm hypodense mass (bracket). An example of an underread and missed finding with only a “prominent” pancreatic duct described in the initial interpretation. Both expert readers independently appreciated this mass with high confidence, with a RADPEER 3 score. The patient was eventually diagnosed with PDAC 6 months later, when she presented with recurrent pain, jaundice and weight loss and abdominal imaging revealed a pancreatic head mass

**Fig. 4:**
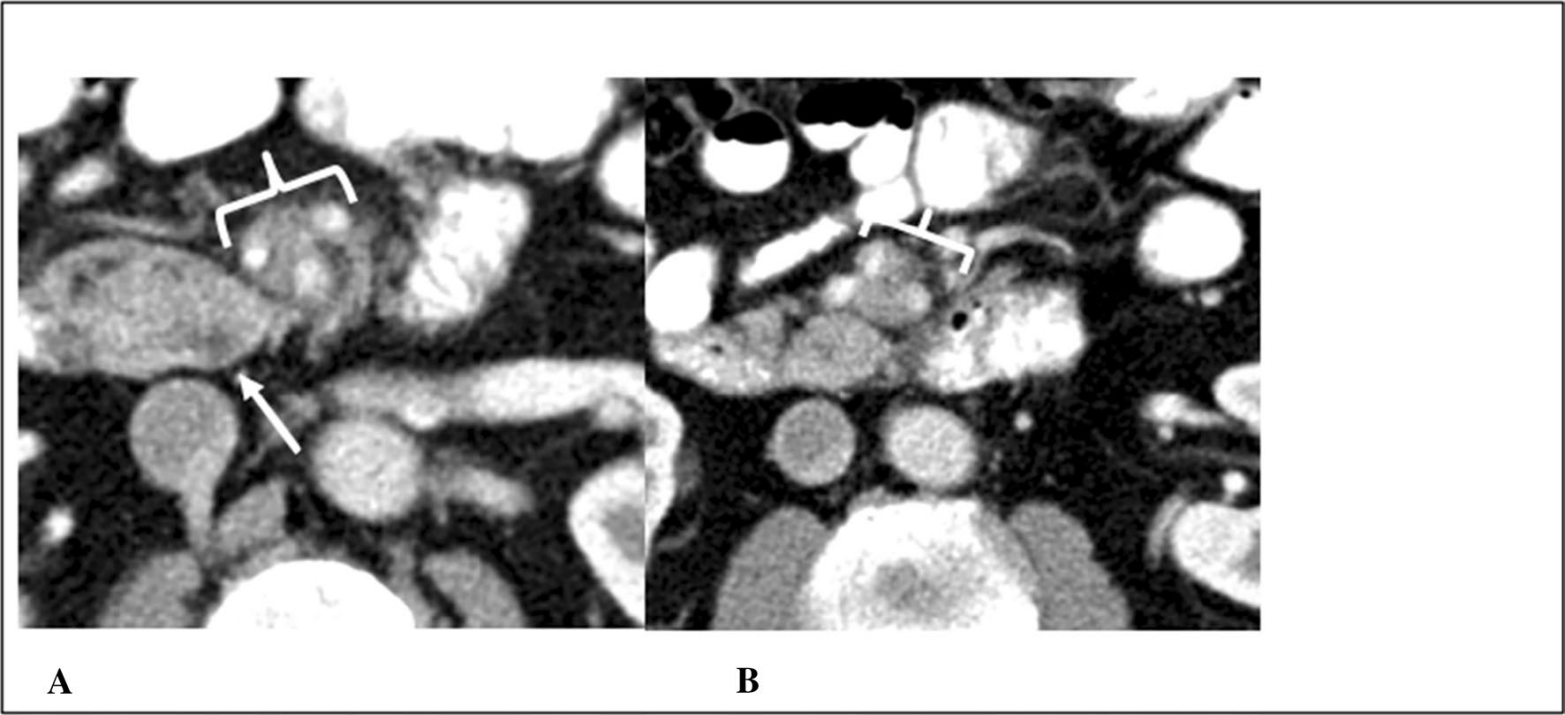
RADPEER 3 score on CT—underread and missed findings. This 69-year old male underwent imaging for evaluation of aspecific abdominal complaints. Axial postcontrast venous phase CT images at the level of the uncinate process (**A**) and 15 mm caudal at the root of the mesentery (**B**). A nearly isodense mass (arrow) is barely discernable, however, more striking is the extensive perivascular soft tissue (brackets) encasing the SMA and SMV to the level of branching in the root of the mesentery. The findings were missed on initial interpretation. Both expert readers independently reported the soft tissues encasement with high confidence. These findings must be treated as an occult mass until proven otherwise

**Fig. 5:**
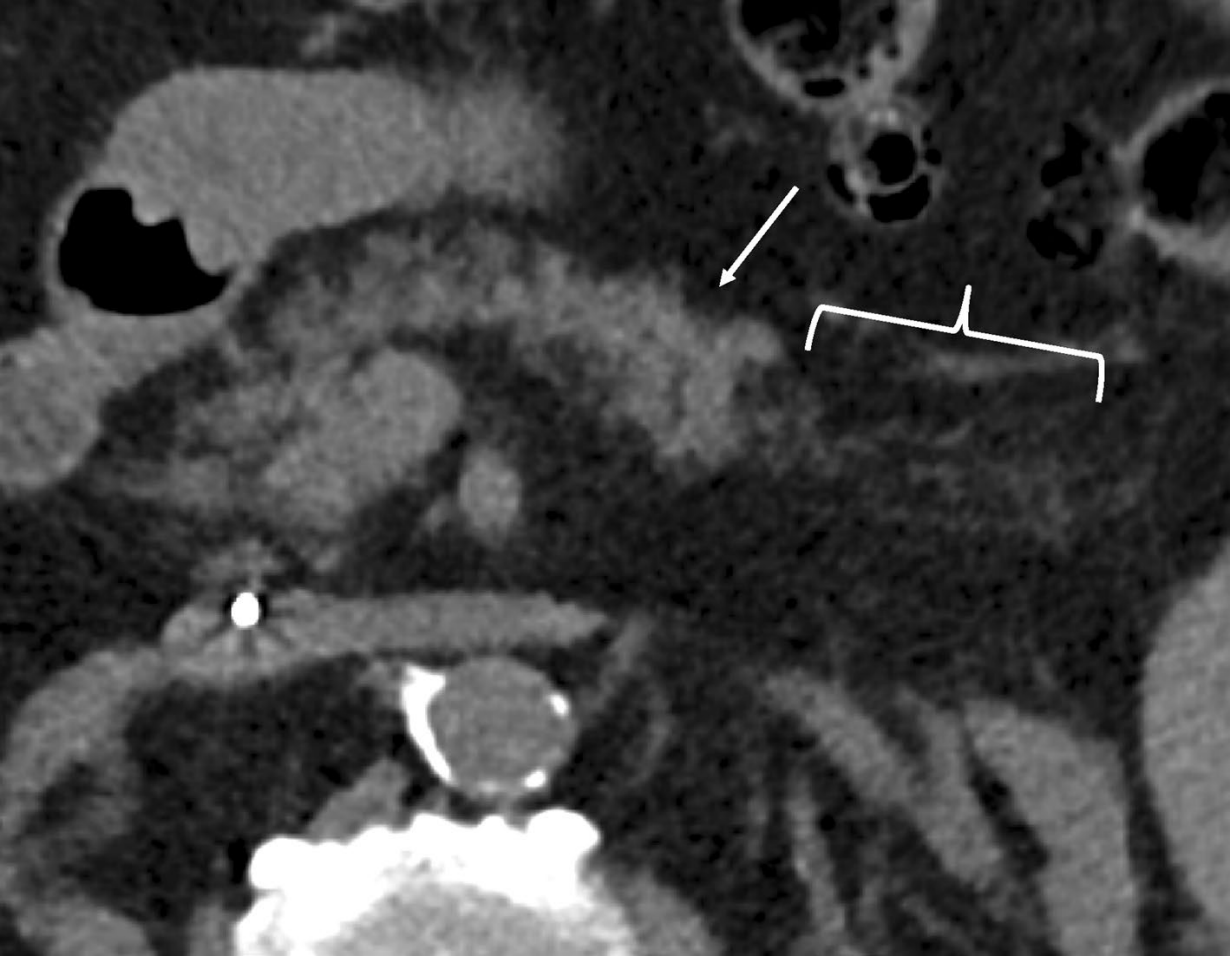
RADPEER 3 score on CT—underread and technical limitations. 82-year old male who underwent a CT abdomen for suspicion of bladder/kidney stones. Axial non-contrast CT (A) demonstrating normal parenchyma in the pancreatic body (arrows) with focal atrophy involving the tail (brackets). The original report noted the pancreas as unremarkable. Both reviewers suspected a pancreatic mass and pancreatic atrophy; reviewer 1 with high confidence and focal tail atrophy, reviewer 2 with low confidence and diffuse atrophy. The patient was subsequently diagnosed with pancreatic cancer in the tail almost 9 months later. This illustrates a technical limitation of non-contrast CT with underreading as a perceptual bias

The original radiology report of 48/60 CTs and 21/27 MRIs were available for evaluation of errors that possibly led to missing or misinterpreting pancreatic abnormalities (Table 4). Technical limitations that may have hindered accurate assessment in CT-imaging included suboptimal slice thickness (≥ 5 mm), non-contrast imaging, single phase imaging, and patient-related factors. For MRI, technical limitations included limited magnet strength (≤ 1.5 T), non-contrast imaging, poor or outdated technique, and motion artifacts. Stratified by the institution of imaging, 10/28 and 9/28 of the available external CT-reports were scored as RADPEER 2 and 3, respectively. Of the internal CT-reports, 3/20 were scored as RADPEER 2 and 4/20 as RADPEER 3. For MRI, 5/5 of the external reports were scored as RADPEER 3, 7/16 internal reports as RADPEER 2 and 3/16 as RADPEER 3. Examples of missed CT-findings are demonstrated in Figs. 3, 4, and 5.

**Table 4:** RADPEER and radiological errors

	CT	MRI
Original report available	48/60	21/27
RADPEER 1, *n* (%)	21 (43.8)	6 (28.6)
RADPEER 2, *n* (%)	14 (29.2)	7 (33.3)
RADPEER 3, *n* (%)	13 (27.0)	8 (38.1)
Time between imaging and diagnosis, months		
RADPEER 1, mean (SD)	15.4 (10.7)	13.7 (7.2)
RADPEER 2, mean (SD)	9.4 (7.5)	9.6 (5.8)
RADPEER 3, mean (SD)	6.6 (3.9)	8.0 (7.8)
Radiological errors		
Underreading, *n* (%)	16 (57.2)	10 (45.5)
Faulty reasoning, *n* (%)	5 (17.9)	1 (4.5)
Satisfaction of search, *n* (%)	3 (10.7)	7 (31.9)
Poor communication, *n* (%)	2 (7.1)	2 (9.1)
Difficult location of lesion, *n* (%)	2 (7.1)	–
Lack of knowledge, *n* (%)	–	1 (4.5)
Complacency, *n* (%)	–	1 (4.5)
Total, *n* (%)	28 (100)	22 (100)
Technical limitations	35/60	15/27

*MRI* magnetic resonance imaging, *CT* computed tomography, *SD* standard deviation

For the 16/21 CT cases that were scored as RADPEER 1 (“concur with original interpretation”), the second unblinded reassessment did not reveal any abnormality of the pancreas. Of the five cases with a retrospective abnormality, 3/5 had pancreatitis and 1/5 had an unsuspicious cystic lesion. In the remaining RADPEER 1 case, the original report mentioned focal PD dilation and mild fullness in the pancreatic head and recommended further evaluation with MRI. Subsequent MRI and EUS were performed and demonstrated signs of chronic pancreatitis, but no distinct mass. Unfortunately, the patient developed jaundice 4 months later and the second EUS demonstrated a clear mass.

**Fig. 6:**
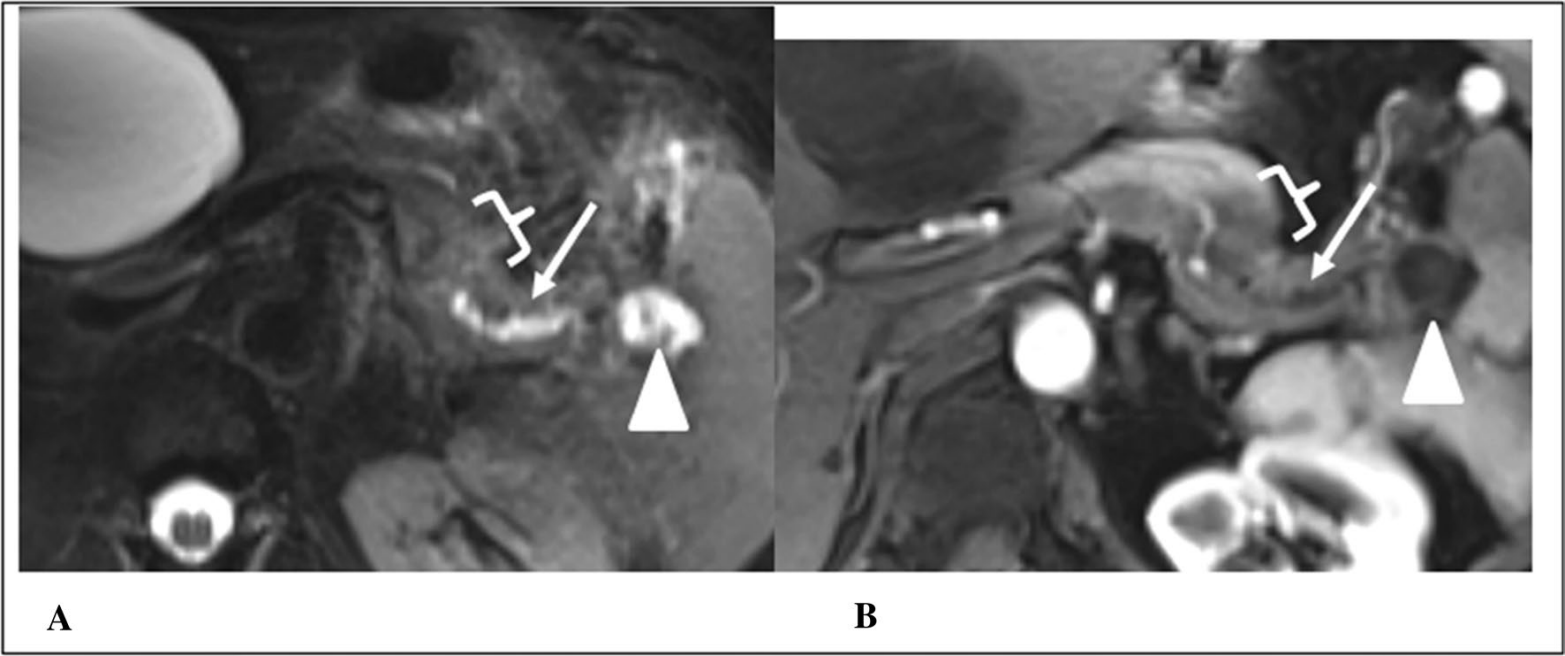
RADPEER 3 score on MRI—missed finding and satisfaction of search. A case of a 63-year old female that underwent an MRI because of a presumed episode of pancreatitis. Axial T2w fat saturated (**A**) and axial T1w fat saturated arterial phase image (**B**) from an outside institution demonstrate pancreatic duct dilation (arrows) and disruption terminating at a 25 mm hypoenhancing tail mass (brackets) with small pseudocyst (arrowhead) in the splenic hilum. The initial interpretation described the pseudocyst and multiple liver cysts; an example of missed finding and satisfaction of search. Both expert readers independently identified the mass and secondary findings with high confidence. The patient presented with recurrent symptoms 8 months later and abdominal imaging revealed a pancreatic tail mass

**Fig. 7:**
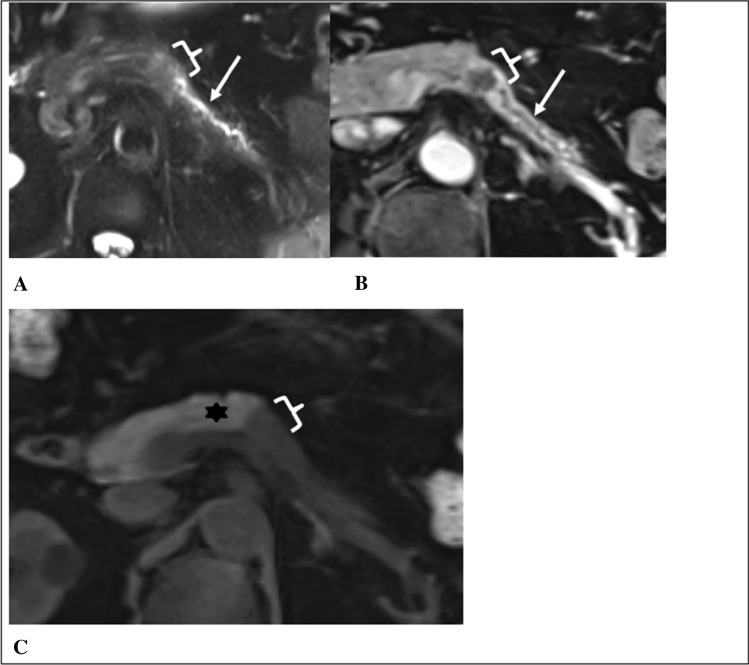
RADPEER 3 score on MRI—misinterpretation. 63-year old male who underwent an MRI because of an accidental finding of hepatomegaly on CT. Axial T2w fat saturated (**A**), axial T1w fat saturated venous phase (**B**), and axial T1w precontrast (**C**) images from an outside institution demonstrate pancreatic duct dilation (arrows) with abrupt terminating at a 25 mm hypoenhancing mass in the body (brackets). T1w precontrast images can be helpful for identifying the demarcation between normal hyperintense parenchyma (asterisks) and hypointense mass with atrophy. On initial report, this lesion was misinterpreted as a "likely benign cyst"; an example of misinterpretation with faulty reasoning and likely lack of knowledge (as a cyst would demonstrate T2 hyperintensity). Both expert readers identified the mass and secondary findings with high confidence. The patient was eventually diagnosed with metastatic pancreatic cancer 4.5 months later

For MRI, 4/6 cases with a RADPEER score of 1 were unremarkable during the blinded and second reassessment. One case was considered normal on the blinded reassessment by both radiologists, however pancreatic duct interruption was questioned upon the unblinded reassessment. Eventually, PDAC was diagnosed 16 months after the MRI. Another RADPEER 1 case had a complex side-branch IPMN in the head of the pancreas for which EUS was recommended in the original report and by both reviewers. Due to altered anatomy after a gastric bypass, the pancreatic head was not completely visualized at that time, and the patient was scheduled for annual follow-up because the cyst had been stable over the preceding year. Nevertheless, the patient presented with severe weight loss 8 months later. MRI then showed abutment of vasculature by the cystic lesion and subsequent surgical resection revealed a 3.2 cm IPMN with invasive adenocarcinoma. Examples of missed and misinterpreted MRI-findings are demonstrated in Figs. 6 and 7.

## Discussion

The study results demonstrate that, in hindsight, a pancreatic mass can already be suspected on pre-diagnostic imaging in 50–70% of patients up to 3 years before PDAC diagnosis. In addition, pancreatic duct dilation, duct interruption, focal atrophy, presence of perivascular soft tissue, and imaging features of acute pancreatitis are strongly associated with pre-diagnostic PDAC, since these features were almost exclusively found in cases and not in age- and gender-matched controls. These results underline that a majority of masses are not (entirely) occult on pre-diagnostic imaging, even if the imaging is performed > 12 months prior to the eventual diagnosis. These results are in agreement with previously reported smaller series [13, 17, 18, 24]. Ahn et al. reported on focal hypoattenuation in all three missed PDAC cases with CT-imaging > 18 months prior to diagnosis, whereas Gangi et al. found PDAC-related features in 50% of patients with imaging 6–18 months prior to diagnosis, but in only 7% of patients with imaging > 18 months prior to diagnosis[17, 18]. Another study found that the mean interval between the first abnormalities seen on MRI in patients with prediagnostic PDAC and the eventual diagnosis was 17 months. In addition, the reviewers in our study were able to detect pancreatic masses with high confidence more frequently on MRI over CT, with the caveat of fewer subjects having pre-diagnostic MRI.

As many as 12% of PDAC patients underwent either an MRI or CT in the pre-diagnostic phase of PDAC, indicating a significant group that could have been diagnosed in an earlier stage of the disease. This study was not designed to assess whether diagnosis at the time of the pre-diagnostic imaging would have led to improved survival. However, more than half of the *visible* masses in this study were staged as T1. Moreover, the results of another recent study on CT-findings in patients with new-onset diabetes who were subsequently diagnosed with PDAC suggested that earlier diagnosis would have led to improved resectability and survival beyond lead time [12].

As stated earlier, PanIN with high-grade dysplasia and early invasive PDAC lesions do not generally form clear hypodense masses. Still, they may cause visible changes of the pancreatic parenchyma and the pancreatic duct, and these changes are rarely observed in patients who do not subsequently develop PDAC, as demonstrated in this study. Focal parenchymal atrophy may be a less known PDAC-related imaging feature, but was observed on CT and MRI in 46%–49% of cases and only in one control patient. These results confirm the conclusion of recently published papers, who recognized focal atrophy as one of the first radiological features of early-stage PDAC [25, 26]. Current practice may underestimate the importance of these secondary findings, especially in the absence of a distinct mass. For patients with PD dilation, interruption, and focal atrophy, thorough examination (e.g., dedicated pancreas imaging with MRI/MRCP, CT or endoscopic ultrasound) and close follow-up is recommended.

Aforementioned secondary findings may be harder to interpret in patients with acute and chronic pancreatitis. Some of the cases in our study had no clinical symptoms of acute pancreatitis, while they did have radiological features of inflammation, emphasizing the importance of confirming imaging findings with the clinical presentation. A previous study demonstrated that an interrupted and dilated PD in the acute setting of pancreatitis was associated with a near-future diagnosis (24 months) of PDAC [27]. Therefore, these features could potentially aid in distinguishing between PDAC-induced pancreatitis and “regular” acute pancreatitis. On the other hand, in the setting of chronic pancreatitis, radiological features like duct dilation and interruption may be less specific for PDAC as investigated in a small case–control study of patients with PDAC and chronic pancreatitis [18]. In their study, PDAC was also suspected in 83% of the patients with chronic pancreatitis, predominantly based on the presence of ductal dilation and interruption.

For research to lead to quality improvement, not only the identification of a problem and its prevalence are important, but also the investigation into the cause of the problem. Discrepancies in interpretation (RADPEER 2 and 3) were more likely to occur in imaging obtained external to our center, particularly in MRI. This finding is in accordance with a meta-analysis on discrepancy rates for body MRI when re-read by a subspecialist [28]. Therefore, re-reading of imaging exams should always be structured and formal, avoiding fixation on externally reported conclusions. Regarding the possible radiological errors that may have hampered accurate detection at the time of imaging, underreading was the most commonly identified error in our study (47% of errors in MRI, 57% in CT), in accordance with previous results [29]. Considering the majority of errors are perceptual, an automatic “red-flag system” using artificial intelligence may improve the incidental detection of early-stage tumors in future practice. Simultaneous imaging interpretation by such a system and the radiologist may point out areas of interest for further investigation, and therefore improve both the accuracy and efficiency of cross-sectional imaging [30].

Although we conducted a comprehensive, matched, case–control study on pre-diagnostic radiological findings of both CT and MRI and presented novel findings, our study may be limited by some factors. To start, our case group had a limited sample size and comprises a heterogeneous group with either CT or MRI with various imaging protocols. Approximately 35% of the reassessed CTs in this study were obtained without contrast, which may have restricted the radiologists’ ability to assess the presence of lesions and secondary signs. This represents a real world scenario, in which unfortunately not all opportunities to detect pre-diagnostic pancreatic cancer will be according to ideal imaging protocols. When pancreas pathology is suspected, the next immediate step after substandard imaging would be to follow-up with the optimal CT and MRI protocols for assessing the pancreas according to society recommendations. Other limitations include the possibility of selection bias as only 12% of PDAC patients had a pre-diagnostic imaging exam available. We acknowledge that the broad utilization of cross-sectional imaging only applies to select countries where these resources are readily available and that early detection on abdominal imaging is not as feasible in areas stressed for resources. Although we have attempted to identify and categorize the radiological errors that may have led to missing or misinterpreting (features of) PDAC, we acknowledge that this is subjectively determined in retrospect.

To conclude, our findings indicate that PDAC-related features on abdominal imaging can be present long before PDAC is diagnosed. These features are rarely present in individuals who are not diagnosed with PDAC, and therefore dedicated pancreas imaging is warranted if these features are found. Future research should focus on an automated second review that can detect otherwise missed lesions or secondary signs, aided by artificial intelligence. In addition, prospective studies should point out if early detection of PDAC would indeed lead to improved survival.

## Supplementary Information

Below is the link to the electronic supplementary material.

Supplementary file1 (DOCX 312 kb)

## Abbreviations

PDAC: Pancreatic ductal adenocarcinoma
PanIN: Pancreatic intraepithelial neoplasia
IPMN: Intraductal pancreatic mucinous neoplasia
CT: Computed tomography
MRI: Magnetic resonance imaging
MRCP: Magnetic resonance cholangio-pancreatography
PACS: Picture archiving and communication system
IQR: Interquartile range
SD: standard deviation

## References

[1] Siegel RL, Miller KD, Jemal A. Cancer statistics, 2020. CA: A Cancer Journal for Clinicians. 2020;70(1):7–30. 10.3322/caac.21590 31912902

[2] Ikemoto J, Serikawa M, Hanada K, Eguchi N, Sasaki T, Fujimoto Y, et al. Clinical Analysis of Early-Stage Pancreatic Cancer and Proposal for a New Diagnostic Algorithm: A Multicenter Observational Study. Diagnostics (Basel). 2021;11(2). 10.3390/diagnostics11020287 PMC7917700 33673151

[3] Blackford AL, Canto MI, Klein AP, Hruban RH, Goggins M. Recent Trends in the Incidence and Survival of Stage 1A Pancreatic Cancer: A Surveillance, Epidemiology, and End Results Analysis. JNCI: Journal of the National Cancer Institute. 2020;112(11):1162–9. 10.1093/jnci/djaa004 PMC7669234 31958122

[4] Matsuda Y, Furukawa T, Yachida S, Nishimura M, Seki A, Nonaka K (2017). The Prevalence and Clinicopathological Characteristics of High-Grade Pancreatic Intraepithelial Neoplasia: Autopsy Study Evaluating the Entire Pancreatic Parenchyma. Pancreas..

[5] Kromrey M-L, Bülow R, Hübner J, Paperlein C, Lerch MM, Ittermann T (2018). Prospective study on the incidence, prevalence and 5-year pancreatic-related mortality of pancreatic cysts in a population-based study. Gut..

[6] Lennon AM, Wolfgang CL, Canto MI, Klein AP, Herman JM, Goggins M (2014). The early detection of pancreatic cancer: what will it take to diagnose and treat curable pancreatic neoplasia?. Cancer Res..

[7] Iacobuzio-Donahue CA (2012). Genetic evolution of pancreatic cancer: lessons learnt from the pancreatic cancer genome sequencing project. Gut..

[8] Yachida S, Iacobuzio-Donahue CA (2013). Evolution and dynamics of pancreatic cancer progression. Oncogene..

[9] Treadwell JR, Zafar HM, Mitchell MD, Tipton K, Teitelbaum U, Jue J. Imaging Tests for the Diagnosis and Staging of Pancreatic Adenocarcinoma: A Meta-Analysis. Pancreas. 2016;45(6). 10.1097/MPA.0000000000000524 26745859

[10] Elbanna KY, Jang H-J, Kim TK (2020). Imaging diagnosis and staging of pancreatic ductal adenocarcinoma: a comprehensive review. Insights into Imaging..

[11] Fukushima D, Nishino N, Hamada K, Horikawa Y, Shiwa Y, Nishida S (2020). Characteristic Radiological Features of Retrospectively Diagnosed Pancreatic Cancers. Pancreas..

[12] Singh DP, Sheedy S, Goenka AH, Wells M, Lee NJ, Barlow J (2020). Computerized tomography scan in pre-diagnostic pancreatic ductal adenocarcinoma: Stages of progression and potential benefits of early intervention: A retrospective study. Pancreatology..

[13] Jang KM, Kim SH, Kim YK, Song KD, Lee SJ, Choi D (2015). Missed pancreatic ductal adenocarcinoma: Assessment of early imaging findings on prediagnostic magnetic resonance imaging. Eur J Radiol..

[14] Pelaez-Luna M, Takahashi N, Fletcher JG, Chari ST (2007). Resectability of presymptomatic pancreatic cancer and its relationship to onset of diabetes: a retrospective review of CT scans and fasting glucose values prior to diagnosis. Am J Gastroenterol..

[15] Kang J, Clarke SE, Abdolell M, Ramjeesingh R, Payne J, Costa AF (2021). The implications of missed or misinterpreted cases of pancreatic ductal adenocarcinoma on imaging: a multi-centered population-based study. European Radiology..

[16] Gonoi W, Hayashi TY, Okuma H, Akahane M, Nakai Y, Mizuno S (2017). Development of pancreatic cancer is predictable well in advance using contrast-enhanced CT: a case–cohort study. European Radiology..

[17] Gangi S, Fletcher JG, Nathan MA, Christensen JA, Harmsen WS, Crownhart BS (2004). Time interval between abnormalities seen on CT and the clinical diagnosis of pancreatic cancer: retrospective review of CT scans obtained before diagnosis. AJR Am J Roentgenol..

[18] Ahn SS, Kim MJ, Choi JY, Hong HS, Chung YE, Lim JS (2009). Indicative findings of pancreatic cancer in prediagnostic CT. Eur Radiol..

[19] Sagami R, Yamao K, Nakahodo J, Minami R, Tsurusaki M, Murakami K (2021). Pre-Operative Imaging and Pathological Diagnosis of Localized High-Grade Pancreatic Intra-Epithelial Neoplasia without Invasive Carcinoma. Cancers..

[20] Hoogenboom SA, Bolan CW, Chuprin A, Raimondo MT, van Hooft JE, Wallace MB (2021). Pancreatic steatosis on computed tomography is an early imaging feature of pre-diagnostic pancreatic cancer: A preliminary study in overweight patients. Pancreatology..

[21] Illuminate S. Illuminate Insight. https://goilluminate.com/solution/insight/; 2021.

[22] Goldberg-Stein S, Frigini LA, Long S, Metwalli Z, Nguyen XV, Parker M (2017). ACR RADPEER Committee White Paper with 2016 Updates: Revised Scoring System, New Classifications, Self-Review, and Subspecialized Reports. J Am Coll Radiol..

[23] Kim YW, Mansfield LT (2014). Fool Me Twice: Delayed Diagnoses in Radiology With Emphasis on Perpetuated Errors. American Journal of Roentgenology..

[24] Kang JD, Clarke SE, Costa AF. Factors associated with missed and misinterpreted cases of pancreatic ductal adenocarcinoma. Eur Radiol. 2020. 10.1007/s00330-020-07307-5 32997176

[25] Miura S, Takikawa T, Kikuta K, Hamada S, Kume K, Yoshida N, et al. Focal Parenchymal Atrophy of the Pancreas Is Frequently Observed on Pre-Diagnostic Computed Tomography in Patients with Pancreatic Cancer: A Case-Control Study. Diagnostics (Basel). 2021;11(9). 10.3390/diagnostics11091693 PMC8471718 34574034

[26] Toshima F, Watanabe R, Inoue D, Yoneda N, Yamamoto T, Sasahira N (2021). CT Abnormalities of the Pancreas Associated With the Subsequent Diagnosis of Clinical Stage I Pancreatic Ductal Adenocarcinoma More Than 1 Year Later: A Case-Control Study. American Journal of Roentgenology..

[27] Abrams D, Mapakshi S, Xu A, Jamali T, Liu Y, Khalaf N. Fr293 RADIOGRAPHIC MARKERS ASSOCIATED WITH NEAR-FUTURE PANCREATIC CANCER DIAGNOSIS AMONG ACUTE PANCREATITIS PATIENTS. Gastroenterology. 2021;160(6):S-286-S-7.

[28] Rosenkrantz AB, Duszak R, Babb JS, Glover M, Kang SK (2018). Discrepancy Rates and Clinical Impact of Imaging Secondary Interpretations: A Systematic Review and Meta-Analysis. J Am Coll Radiol..

[29] Bruno MA, Walker EA, Abujudeh HH (2015). Understanding and Confronting Our Mistakes: The Epidemiology of Error in Radiology and Strategies for Error Reduction. RadioGraphics..

[30] Conant EF, Toledano AY, Periaswamy S, Fotin SV, Go J, Boatsman JE, et al. Improving Accuracy and Efficiency with Concurrent Use of Artificial Intelligence for Digital Breast Tomosynthesis. Radiology: Artificial Intelligence. 2019;1(4):e180096. 10.1148/ryai.2019180096 PMC6677281 32076660

