# Supplementary material for: Prevalence, features, and explanations of missed and misinterpreted pancreatic cancer on imaging: a matched case–control study
Source: Abdom Radiol (NY). 2022 Sep 21;47(12):4160–72. doi: 10.1007/s00261-022-03671-6 (PMC9626431; doi:10.1007/s00261-022-03671-6)
Supplement: Supplementary file 1 — Supplementary file1 (DOCX 312 kb) [file 261_2022_3671_MOESM1_ESM.docx]

# **Supplemental Material**

**Supplemental material 1.**

## **Imaging analysis – CT - Blinded review of missed and misinterpreted features**

- CT with or without contrast
- Pancreas normal? 1 = yes, 0 = no
- Greatest CBD diameter (mm)
- Acute pancreatitis? 1 = yes, 0 = no
  - If AP, location 0 = uncinate 1 = head 2 = body 3 = tail 4= diffuse 99 = Unknown 88 = Not applicable
- Chronic pancreatitis? 1 = yes, 0 = no
  - If CP, severity according to Cambridge classification (equivocal = 1, mild = 2, moderate = 3, severe = 4)
- Pancreatic Cystic lesion (PCL)? 1 = yes, 0 = no
  - If PCL, location 0 = uncinate 1 = head 2 = body 3 = tail 4= diffuse 99 = Unknown 88 = Not applicable
  - If PCL, define most likely type FREE TEXT 99 = Unknown 88 = Not applicable
- Pancreatic mass? 1 = yes, 0 = no
  - If mass, confidence level 1 = high, 0 = low
  - If mass, location 0 = uncinate 1 = head 2 = body 3 = tail 4= diffuse 99 = Unknown 88 = Not applicable
  - If mass, size (mm) greatest axial
- Greatest PD diameter (mm)
  - If dilated (> 3 mm), location greatest PD diameter 0 = uncinate 1 = head 2 = body 3 = tail 4= diffuse 99 = Unknown 88 = Not applicable
- Disrupted PD?
  - Location disrupted PD 0 = uncinate 1 = head 2 = body 3 = tail 4= diffuse 99 = Unknown 88 = Not applicable
- Parenchymal atrophy? 1 = yes, 0 = no
  - If atrophy, location: 0 = uncinate 1 = head 2 = body 3 = tail 4= diffuse 99 = Unknown 88 = Not applicable
  - Enhancement pancreas venous or pancreas phase? 0 = hypo 1 = iso 2 = hyper 88 = N/A 99 = unknown
- Perivascular soft tissue 1=yes, 0=no
- Recommend additional imaging for within 4 weeks 1 = yes, 0 = no
- Recommend additional imaging, but later than 4 weeks 1 = yes, 0 = no
- Comments

## **Imaging analysis – MRI - Blinded review of missed and misinterpreted features**

- MRI protocol
- Pancreas normal? 1 = yes, 0 = no
- Greatest CBD diameter (mm)
- Acute pancreatitis? 1 = yes, 0 = no
  - If AP, location 0 = uncinate 1 = head 2 = body 3 = tail 4= diffuse 99 = Unknown 88 = Not applicable
- Chronic pancreatitis? 1 = yes, 0 = no
  - If CP, severity according to Cambridge classification (equivocal = 1, mild = 2, moderate = 3, severe = 4)
- Pancreatic Cystic lesion (PCL)? 1 = yes, 0 = no
  - If PCL, location 0 = uncinate 1 = head 2 = body 3 = tail 4= diffuse 99 = Unknown 88 = Not applicable
  - If PCL, define most likely type FREE TEXT 99 = Unknown 88 = Not applicable
- Pancreatic mass? 1 = yes, 0 = no
  - If mass, confidence level 1 = high, 0 = low
  - If mass, location 0 = uncinate 1 = head 2 = body 3 = tail 4= diffuse 99 = Unknown 88 = Not applicable
  - If mass, size (mm) greatest axial
- Greatest PD diameter (mm)
  - If dilated (> 3 mm), location greatest PD diameter 0 = uncinate 1 = head 2 = body 3 = tail 4= diffuse 99 = Unknown 88 = Not applicable
- Disrupted PD?
  - Location disrupted PD 0 = uncinate 1 = head 2 = body 3 = tail 4= diffuse 99 = Unknown 88 = Not applicable
- Parenchymal atrophy? 1 = yes, 0 = no
  - If atrophy, location: 0 = uncinate 1 = head 2 = body 3 = tail 4= diffuse 99 = Unknown 88 = Not applicable
- Enhancement pancreas venous or pancreas phase? 0 = hypo 1 = iso 2 = hyper 88 = N/A 99 = unknown
- T1 signal lesion? 0 = hypo, 1 = iso, 2 = hyper
- T2 signal lesion? 0 = hypo, 1 = iso, 2 = hyper
- DWI sequences available? 1 = yes, 0 = no
- DWI on 800 lesion? restriction 1 = yes 0 = no
- Most conspicuous on which sequence? Free text
- Recommend additional imaging for within 4 weeks 1 = yes, 0 = no
- Recommend additional imaging, but later than 4 weeks 1 = yes, 0 = no
- Comments

## **Second reassessment – Unblinded RADPEER evaluation**

*Reassessment factors in bold below were provided prior to the Radiologist’s unblinded evaluation.
Reassessment factors not in bold were scored by the Radiologist during the unblinded evaluation.*

- **Modality (CT or MR)**
  - If MRI: DWI available?
- **Months between imaging and diagnosis**
- **Indication of imaging**
- **Reassessment Reviewer 1 (most serious finding)**
- **Reassessment Reviewer 2 (most serious finding)**
- **Original report**
- **Location PDAC (eventual diagnosis)**
- Unblinded reassessment: signs of a mass?
- RADPEER score:
  - 1 = concur with interpretation;
  - 2 = discrepancy in interpretation but “an understandable miss";
  - 3 = discrepancy in interpretation that would have been expected to be made
  - + a/b: a = unlikely to be clinically significant; b = likely to be clinically significant.
- Technical limitations (CT: usage of contrast, slice thickness, protocol, MRI: Magnet strength, protocol, DWI). Patient factors: motion, obesity.
- Perceptual bias (incl. underreading, location, satisfaction of search).
- Cognitive bias (incl. complacency, faulty reasoning, lack of knowledge, communication, prior examination, history, complication, satisfaction of report).
- If suspected tumor on pre-diagnostic CT, TNM stage
- **If suspected tumor, size (mm)**
- When size not in agreement, final measurement (mm)
- Comments (for example, illustrative example CT/MR, please include series and slice number

**Supplemental material 2.**

| **Indications for CT-imaging** | **Cases (n=60)** | **Controls (n=235)** |
| --- | --- | --- |
| - (Follow-up) hepatobiliary disease, n (%) | 2 (3.3) | 16 (6.8) |
| - (Follow-up) pancreatic disease, n (%) | 9 (15.0) | 1 (0.4) |
| - - Cystic lesion, n (%) | 3 (5.0) | - |
| - - Chronic/acute pancreatitis, n (%) | 5 (8.3) | 1 (0.4) |
| - - Steatorrhea | 1 (1.7) | - |
| - Follow-up cancer, n (%) | 12 (20.0) | 42 (17.9) |
| - (Follow-up) renal or urinary tract disease, n (%) | 4 (6.7) | 74 (31.5) |
| - Abdominal pain, n (%) | 24 (40.0) | 67 (28.5) |
| - Weight loss, n (%) | 1 (1.7) | 3 (1.3) |
| - (Follow-up) vascular disease, n (%) | 1 (1.7) | 17 (7.2) |
| - Other, n (%) | 2 (3.3) | 15 (6.4) |
| - Missing, n (%) | 5 (8.3) | - |
| Total | 60 | 235 |

**Supplemental material 3.**

| **Indications for MR-imaging** | **Cases (n = 27)** | **Controls (n = 103)** |
| --- | --- | --- |
| - (Follow-up) hepatobiliary disease, n (%) | 8 (29.6) | 67 (65.0) |
| - (Follow-up) pancreatic disease, n (%) | 12 (44.4) | 7 (6.8) |
| - - Cystic lesion, n (%) | - 6 (22.2) | - 5 (4.9) |
| - - Chronic/acute pancreatitis, n (%) | - 4 (14.8) | - 1 (1.0) |
| - - Abnormality pancreas, n (%) | - 2 (7.4) | - 1 (1.0) |
| - Follow-up cancer, n (%) | 2 (7.4) | 13 (12.6) |
| - (Follow-up) renal disease, n (%) | 1 (3.7) | 8 (7.8) |
| - Abdominal pain, n (%) | 4 (14.8) | 4 (3.9) |
| - (Follow-up) vascular disease, n (%) | - | 3 (2.9) |
| - Other, n (%) | - | 1 (1.0) |
| Total | 27 | 103 |

**Supplemental material 4**

| **Classification of radiological errors** |
| --- |
| 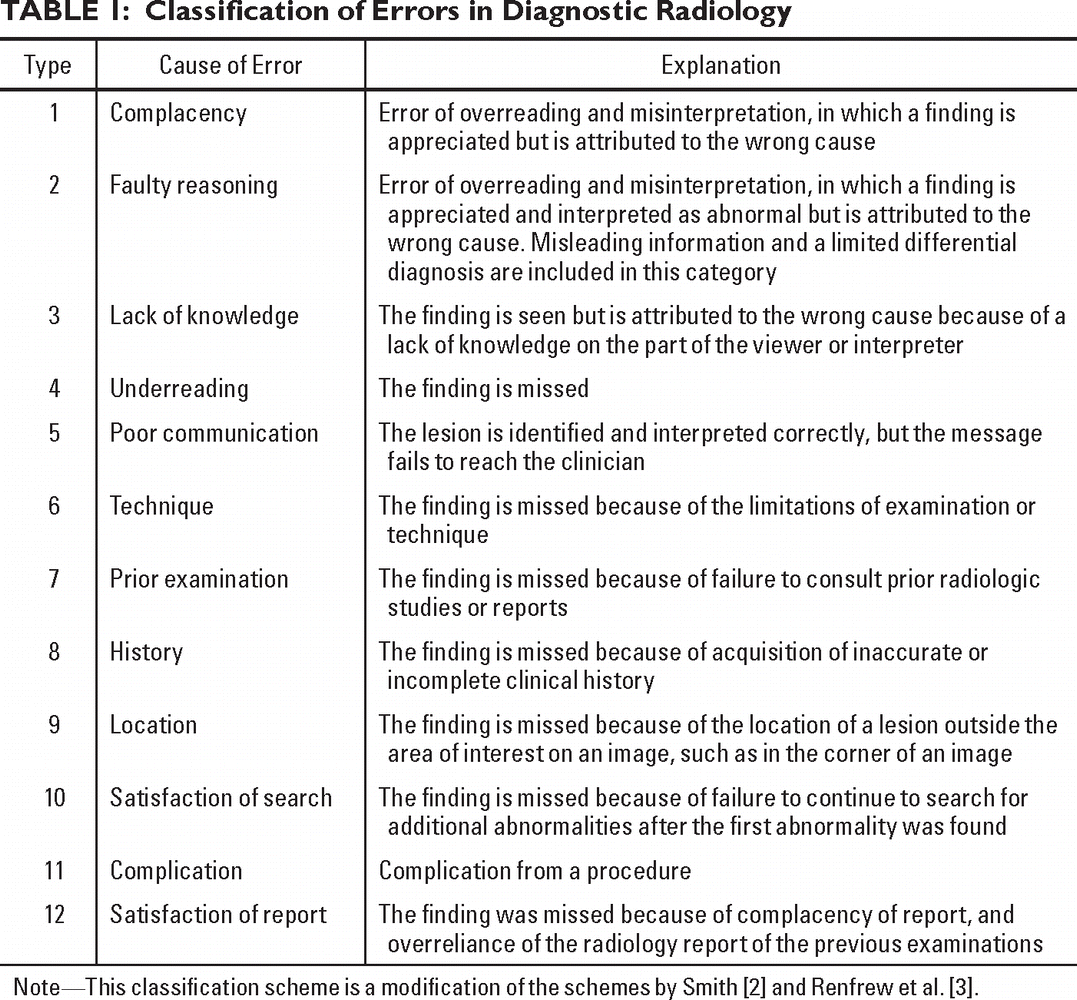 |
| Reprinted with permission from ‘Fool Me Twice: Delayed Diagnoses in Radiology With Emphasis on Perpetuated Errors’, Young W. Kim and Liem T. Mansfield [23], the American Journal of Roentgenology, 202/3, Copyright© 2014, American Roentgen Ray Society, ARRS, All Rights Reserved. |
